# Review: regulatory mechanisms of gonadotropin-inhibitory hormone (GnIH) synthesis and release in photoperiodic animals

**DOI:** 10.3389/fnins.2013.00060

**Published:** 2013-04-16

**Authors:** Kazuyoshi Tsutsui, Takayoshi Ubuka, George E. Bentley, Lance J. Kriegsfeld

**Affiliations:** ^1^Laboratory of Integrative Brain Sciences, Department of Biology, Waseda University, and Center for Medical Life Science of Waseda UniversityTokyo, Japan; ^2^Department of Integrative Biology, Helen Wills Neuroscience Institute, University of CaliforniaBerkeley, Berkeley, CA, USA; ^3^Department of Psychology, Helen Wills Neuroscience Institute, University of CaliforniaBerkeley, Berkeley, CA, USA

**Keywords:** gonadotropin-releasing hormone (GnRH), gonadotropin-inhibitory hormone (GnIH), gonadotropins, melatonin, glucocorticoids, photoperiod, stress, reproduction

## Abstract

Gonadotropin-inhibitory hormone (GnIH) is a novel hypothalamic neuropeptide that was discovered in quail as an inhibitory factor for gonadotropin release. GnIH inhibits gonadotropin synthesis and release in birds through actions on gonadotropin-releasing hormone (GnRH) neurons and gonadotropes, mediated via the GnIH receptor (GnIH-R), GPR147. Subsequently, GnIH was identified in mammals and other vertebrates. As in birds, mammalian GnIH inhibits gonadotropin secretion, indicating a conserved role for this neuropeptide in the control of the hypothalamic-pituitary-gonadal (HPG) axis across species. Identification of the regulatory mechanisms governing GnIH expression and release is important in understanding the physiological role of the GnIH system. A nocturnal hormone, melatonin, appears to act directly on GnIH neurons through its receptor to induce expression and release of GnIH in quail, a photoperiodic bird. Recently, a similar, but opposite, action of melatonin on the inhibition of expression of mammalian GnIH was shown in hamsters and sheep, photoperiodic mammals. These results in photoperiodic animals demonstrate that GnIH expression is photoperiodically modulated via a melatonin-dependent process. Recent findings indicate that GnIH may be a mediator of stress-induced reproductive disruption in birds and mammals, pointing to a broad role for this neuropeptide in assessing physiological state and modifying reproductive effort accordingly. This paper summarizes the advances made in our knowledge regarding the regulation of GnIH synthesis and release in photoperiodic birds and mammals. This paper also discusses the neuroendocrine integration of environmental signals, such as photoperiods and stress, and internal signals, such as GnIH, melatonin, and glucocorticoids, to control avian and mammalian reproduction.

## Introduction

The neuroendocrine integration of environmental and internal signals controls reproduction across vertebrate species. The reproductive axis of vertebrates integrates information from a wide range of systems via direct and indirect neurochemical inputs. Many of the neuropeptidergic pathways involved in the transduction of environmental stimuli into neuroendocrine signals have been well-studied. A hypothalamic neuropeptide, gonadotropin-releasing hormone (GnRH), is the primary factor regulating secretion of both of the gonadotropins, luteinizing hormone (LH), and follicle-stimulating hormone (FSH), and acts as a key neurohormone for vertebrate reproduction. Since the discovery of GnRH in the brain of mammals at the beginning of 1970s (Matsuo et al., 1971; Burgus et al., 1972), several other GnRHs have been identified in the brain of a variety of vertebrates (King and Millar, 1982; Miyamoto et al., 1982, 1984; Sherwood et al., 1983, 1986; Jimenez-Liñan et al., 1997; Okubo et al., 2000a,b; Yoo et al., 2000). In contrast, an inhibitory hypothalamic neuropeptide for gonadotropin secretion was, until recently, unknown, although gonadal sex steroids and inhibin can modulate gonadotropin secretion. Findings from the last 12 years, however, indicate that GnRH is not the sole hypothalamic regulatory neuropeptide of vertebrate reproduction, with gonadotropin-inhibitory hormone (GnIH) playing a key role in the inhibition of reproduction.

In 2000, GnIH was discovered in the avian brain. In a search for novel neuropeptides regulating the release of pituitary hormones, Tsutsui and colleagues identified a novel hypothalamic neuropeptide that directly acts on the pituitary to inhibit gonadotropin release in quail and termed it GnIH (Tsutsui et al., 2000). This was the first demonstration of a hypothalamic neuropeptide inhibiting gonadotropin release in any vertebrate. From the past 12 years of research, we now know that GnIH exists in a variety of avian species and regulates avian reproduction by decreasing gonadotropin release and synthesis via action on the anterior pituitary gland and the GnRH system, mediated via the GnIH receptor (GnIH-R), GPR147 (Tsutsui et al., 2000, 2005, 2006, 2007a,b, 2010a,b, 2012; Satake et al., 2001; Bentley et al., 2003, 2006, 2008, 2010; Ubuka et al., 2003, 2005, 2006, 2008a,b, 2009a,b, 2012a,b; Ukena et al., 2003; Ciccone et al., 2004; Osugi et al., 2004; Yin et al., 2005; Tsutsui and Ukena, 2006; Tsutsui, 2009; Tobari et al., 2010; Tsutsui and Ubuka, 2013).

Tsutsui and colleagues have further identified GnIH orthologs in other vertebrates including mammals (for reviews, see Ukena and Tsutsui, 2005; Tsutsui and Ukena, 2006; Tsutsui et al., 2007a, 2010a,b; Tsutsui, 2009; Kriegsfeld et al., 2010; Smith and Clarke, 2010; Tsutsui and Ubuka, 2013). As in birds, mammalian GnIH orthologs appear to act as key neurohormones to inhibit gonadotropin release in several mammalian species (Kriegsfeld et al., 2006; Johnson et al., 2007; Clarke et al., 2008; Gibson et al., 2008; Murakami et al., 2008; Kadokawa et al., 2009; Sari et al., 2009; Ubuka et al., 2012a). Furthermore, fish GnIH orthologs also inhibit gonadotropin release in fish (Zhang et al., 2010; Moussavi et al., 2012), indicating a conserved role for GnIH and its orthologs in the control of the hypothalamo-pituitary-gonadal (HPG) axis across species. Thus, GnIH, a newly discovered hypothalamic neuropeptide, and its orthologs appear to act as a key neurohormone controlling vertebrate reproduction, generally (for reviews, see Tsutsui, 2009; Tsutsui et al., 2010a,b).

Following the discovery of GnIH, kisspeptin, encoded by the *Kiss1* gene, was discovered in mammals. In contrast to GnIH, kisspeptin has a stimulatory effect on GnRH neurons via its receptor, GPR54, causing up-regulation of the HPG axis (for reviews, see Ukena and Tsutsui, 2005; Tsutsui and Ukena, 2006; Tsutsui, 2009; Tsutsui et al., 2010a). Kisspeptin and GPR54 are considered to be essential for puberty and subsequent fertility in mammals. At present, the *Kiss1* gene has been identified in most vertebrates, including mammals, amphibians, and fish (for reviews, see Ukena and Tsutsui, 2005; Tsutsui and Ukena, 2006; Tsutsui, 2009; Tsutsui et al., 2010a). Most recently, we found a second isoform of *Kiss1*, designated *Kiss2*, in several vertebrate groups, but not in birds and rodents (Lee et al., 2009).

Thus, the discovery of GnIH and kisspeptin has changed our understanding of the vertebrate reproductive axis despite the well-accepted dogma that reproduction is mainly under stimulatory control from GnRH. Seasonal breeders such as photoperiodic animals rely on environmental and internal cues to initiate and/or terminate reproduction, and these signals need to be further translated and integrated to generate the appropriate neuroendocrine response. Herein we review what is currently known about the regulation of GnIH synthesis and release by photoperiods and melatonin, a pineal hormone, in photoperiodic birds and mammals. We also highlight the effect of stress on GnIH synthesis. Finally, we discuss the neuroendocrine integration of environmental signals, such as photoperiods and stress, and internal signals, such as GnIH, melatonin, and glucocorticoids, to control avian and mammalian reproduction.

## Structure and function

**Figure 1:**
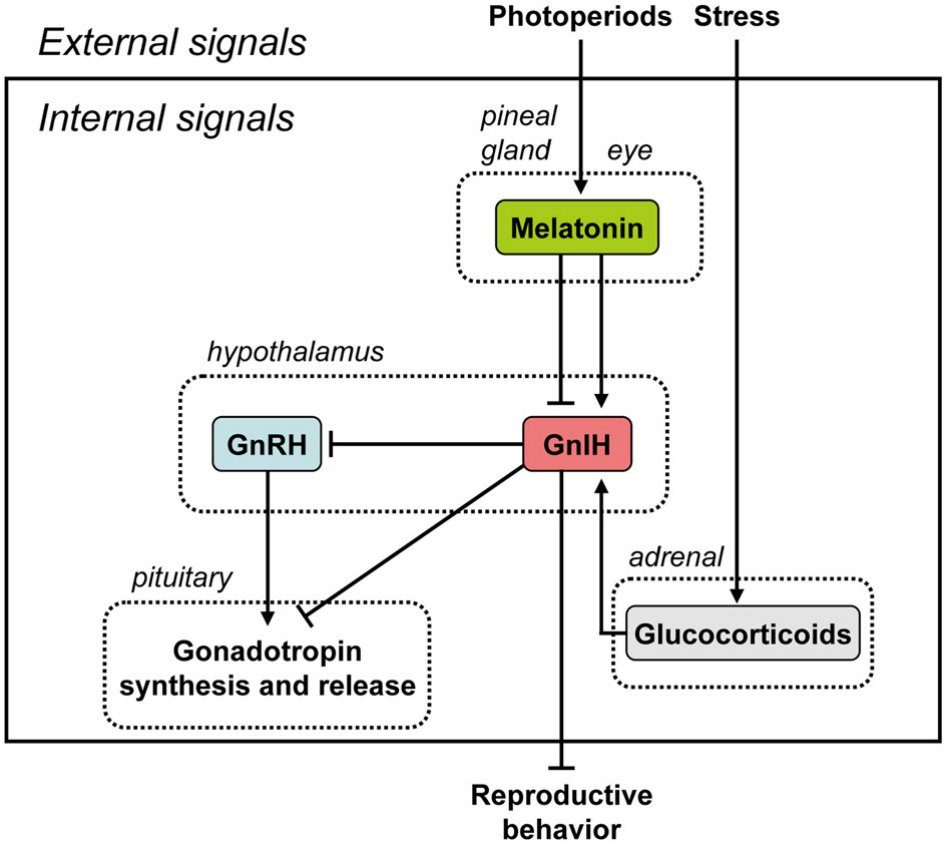
**Summary of the neuroendocrine integration of environmental signals and internal signals to control reproduction.** The neuroendocrine integration of environmental signals, such as photoperiods and stress, and internal signals, such as GnIH, melatonin, and glucocorticoids, is important for the control of avian and mammalian reproduction. GnIH inhibits gonadotropin synthesis and release by directly acting on the pituitary or by inhibiting the activity of GnRH neurons in birds and mammals. GnIH can also inhibit reproductive behavior by possibly acting within the brain. GnIH expression is photoperiodically modulated via a melatonin-dependent process. Melatonin induces the expression of GnIH in quail and rats, whereas melatonin inhibits the expression of GnIH in hamsters and sheep. Stress induces the expression of GnIH in birds and mammals. GnIH may therefore be a mediator of stress-induced reproductive disruption. See the text for details.

It was believed for a long time that GnRH is the only hypothalamic neuropeptide regulating pituitary gonadotropin synthesis and release in vertebrates. In 2000, however, GnIH was discovered in the brain of the Japanese quail whilst in search of a novel hypothalamic neuropeptide having a C-terminal Arg-Phe-NH_2_ motif (RFamide peptide) (Tsutsui et al., 2000). GnIH has a previously-unreported dodecapeptide structure, Ser-Ile-Lys-Pro-Ser-Ala-Tyr-Leu-Pro-Leu-Arg-Phe-NH_2_ (SIKPSAYLPLRFamide) (Tsutsui et al., 2000). Its C-terminus is identical to chicken LPLRFamide that was reported to be the first RFamide peptide isolated in vertebrates (Dockray et al., 1983), which is likely to be a degraded fragment of GnIH (for reviews, see Ukena and Tsutsui, 2005; Tsutsui, 2009; Tsutsui et al., 2010a,b). After the isolation of GnIH in the quail brain, the precursor polypeptide for GnIH was examined (Satake et al., 2001). A cDNA that encodes GnIH precursor polypeptide was identified by a combination of 3′ and 5′ rapid amplification of cDNA ends (3′/5′ RACE) in quail (Satake et al., 2001) and other avian species, such as chickens, sparrows, zebra finches, and starlings (for reviews, see Tsutsui, 2009; Tsutsui et al., 2010a,b). The GnIH precursor encodes one GnIH and two GnIH-related peptides (GnIH-RP-1 and GnIH-RP-2) possessing an LPXRFamide (X = L or Q) sequence at their C-termini in all avian species studied. GnIH was further isolated as a mature peptide in starlings (Ubuka et al., 2008a) and zebra finches (Tobari et al., 2010) and GnIH-RP-2 was also identified in quail (Satake et al., 2001). From the past 12 years of research, thus we now know that GnIH exists in various avian species, and regulates avian reproduction by decreasing gonadotropin release and synthesis via action on gonadotropes in the anterior pituitary gland and GnRH neurons in the hypothalamus, mediated via the GnIH-R, GPR147 (Tsutsui et al., 2000, 2005, 2006, 2007a,b, 2010a,b; Satake et al., 2001; Bentley et al., 2003, 2006, 2010; Ubuka et al., 2003, 2005, 2006, 2008a,b, 2009a,b, 2012a,b; Ukena et al., 2003; Ciccone et al., 2004; Osugi et al., 2004; Yin et al., 2005; Tsutsui and Ukena, 2006; Tsutsui, 2009; Tobari et al., 2010; Tsutsui and Ubuka, 2013) (Figure 1).

After the discovery of GnIH in birds, GnIH orthologs have been further identified in a number of other vertebrates including mammals (for reviews, see Tsutsui et al., 2007a, 2010a,b; Tsutsui, 2009). In mammals, cDNAs that encode LPXRFamide peptides similar to GnIH have been investigated by a gene database search (Hinuma et al., 2000). The cDNAs identified from mammalian brain encode three GnIH orthologs [known as RFamide-related peptides (RFRPs)], RFRP-1, -2, and -3, in bovines and humans and two GnIH orthologs, RFRP-1 and -3, in rodents (for reviews, see Tsutsui, 2009; Tsutsui et al., 2010a,b). RFRP-1 and -3 are both LPXRFamide peptides, but RFRP-2 is not an LPXRFamide peptide. The mammalian GnIH orthologs, RFRP-1 and/or RFRP-3 were also identified as mature peptides in bovines (Fukusumi et al., 2001; Yoshida et al., 2003), rats (Ukena et al., 2002), hamsters (Ubuka et al., 2012a), monkeys (Ubuka et al., 2009a), and humans (Ubuka et al., 2009b). Subsequently, it was shown that RFRP-3 inhibits gonadotropin synthesis and/or release in various mammalian species (Johnson et al., 2007; Clarke et al., 2008; Murakami et al., 2008; Kadokawa et al., 2009; Sari et al., 2009; Ubuka et al., 2012a). In addition, RFRP-3 was shown to inhibit GnRH-stimulated gonadotropin synthesis in mammalian pituitary gonadotropes (Sari et al., 2009). Accordingly, at least RFRP-3 acts as a functional ortholog of GnIH in terms of reducing gonadotropin secretion (for reviews, see Tsutsui, 2009; Tsutsui et al., 2010a,b). More recently, it was found that intracerebroventricular (ICV) administration of RFRP-1 also inhibits gonadotropin release in hamsters (Ubuka et al., 2012a). Thus, as in birds, the mammalian GnIH orthologs, RFRP-1 and RFRP-3, act to inhibit gonadotropin secretion in mammals (Kriegsfeld et al., 2006, 2010; Johnson et al., 2007; Clarke et al., 2008; Gibson et al., 2008; Murakami et al., 2008; Kadokawa et al., 2009; Ubuka et al., 2012a). Recently, an inhibitory action of fish GnIH orthologs (LPXRFamide peptides) on gonadotropin release was also reported in zebrafish (Zhang et al., 2010) and goldfish (Moussavi et al., 2012). In general, GnIH and its orthologs seem to act similarly across vertebrate species to regulate reproduction, although some exceptions exist.

## Localization and mode of action

In birds, GnIH neurons are located in the paraventricular nucleus (PVN) in the hypothalamus and their major projections reach the median eminence of the hypothalamus (Tsutsui et al., 2000; Ubuka et al., 2003; Ukena and Tsutsui, 2005). Based on the observation that GnIH-immunoreactive (-ir) neurons project to the external layer of the median eminence in quail, the first study on GnIH (Tsutsui et al., 2000) focused on the effect of GnIH on pituitary gonadotropin release. In songbirds, intravenous (IV) injection of GnIH can rapidly reduce circulating LH in breeding white-crowned sparrows in addition to inhibiting GnRH-induced LH release in non-breeding song sparrows (Osugi et al., 2004). GnIH administration *in vivo* and *in vitro* inhibits the synthesis of LHβ- and FSHβ-subunits within the pituitary gland of quail and chickens (Ciccone et al., 2004; Ubuka et al., 2006), indicating a dual role for GnIH within the pituitary-acting over different time-frames to reduce first the release of gonadotropins into the circulation followed by inhibition of LH and FSH synthesis. Thus, it has become clear that GnIH in birds is an important regulator of pituitary gonadotropin synthesis in addition to gonadotropin release (Ciccone et al., 2004; Osugi et al., 2004; Bentley et al., 2006; Ubuka et al., 2006) (Figure 1). Despite our published data on the distribution of GnIH in the median eminence and the GnIH-R, GPR147 in the pituitary, there are some inconsistencies in the literature. For example, rufous-winged sparrows do not seem to exhibit GnIH-ir fibers in the median eminence (Small et al., 2007). Nor do IV injections of GnIH rapidly inhibit LH secretion in this species (Deviche et al., 2006). It may be possible that there is another source of GnIH that can influence pituitary gonadotropin release.

The binding activity of GnIH-R was characterized in the quail (Yin et al., 2005). The crude membrane fraction of COS-7 cells transfected with the putative GnIH-R (GPR147) cDNA specifically bound GnIH and GnIH-RPs in a concentration-dependent manner, indicating that GPR147 is GnIH-R (Yin et al., 2005). GPR147 is a member of the G-protein coupled receptor (GPCR) family which couples to Gα and, upon activation inhibits adenylyl cyclase (AC) activity, thus reducing intracellular cAMP levels (Bédécarrats et al., 2009; Shimizu and Bédécarrats, 2010). Type III GnRH receptor (GnRH-R-III) is the pituitary specific GnRH receptor in the chicken (Shimizu and Bédécarrats, 2006). In the chicken pituitary gland, the chicken GnRH-R-III (cGnRH-R-III)/GnIH-R ratio changes during sexual maturation in favor of cGnRH-R-III that appears to result in hypothalamic control of gonadotropin secretion shifting from inhibitory to stimulatory, with corresponding changes in GnRH-induced cAMP levels (Shimizu and Bédécarrats, 2010). GnIH-R mRNA quantity was significantly higher in the pituitaries of sexually immature chickens relative to sexually mature chickens (Maddineni et al., 2008). Estradiol or a combination of estradiol and progesterone treatment caused a significant decrease in pituitary GnIH-R mRNA (Maddineni et al., 2008). Furthermore, GnIHR-ir cells were found to be colocalized with LHβ mRNA-, or FSHβ mRNA-containing cells, possibly mediating the inhibitory effect of GnIH on LH and FSH secretion (Maddineni et al., 2008) (Figure 1).

In addition to the effects of GnIH on the pituitary, the contact of GnRH neurons by GnIH axons is perhaps the most conserved property of GnIH:GnRH interactions in birds and other vertebrates (Figure 1). Initially discovered in house sparrows (Bentley et al., 2003), contact of GnRH neurons by GnIH has been observed in all other vertebrates studied to date, including humans (Ubuka et al., 2009b). In birds, GnIH neurons project to GnRH-I and -II neurons and presumably inhibit the action of these two types of GnRH via the GnIH-R, GPR147, in European starlings (Ubuka et al., 2008a). Experimental support of this notion comes from Bentley et al. (2006), in which centrally-infused GnIH inhibited circulating LH and reduced copulation solicitation in female white-crowned sparrows. Further, rhodaminated GnIH was shown to bind to putative GnRH-II neurons and, in a later study on European starlings, GnRH-I and -II neurons were shown to express GnIH-R (GPR147) mRNA (Ubuka et al., 2008a). Thus, physiological, behavioral and histological evidence in several species combine to indicate a direct role for GnIH in regulation of GnRH in the brain of songbirds (Figure 1). In contrast to the highly-clustered cell bodies in the PVN, GnIH-ir fibers are widely distributed in diencephalic and mesencephalic regions in birds, suggesting the regulation of other, non-reproductive behaviors (Bentley et al., 2003; Ukena et al., 2003; Ubuka et al., 2008a). For example, central administration of GnIH stimulates feeding behavior in chickens (Tachibana et al., 2005).

To identify the mechanism of GnIH neurons in the regulation of behavior, Ubuka et al. (2012b) investigated the effect of RNA interference (RNAi) of the GnIH gene on the behavior of white-crowned sparrows, a highly social songbird species. Administration of small interfering RNA against GnIH precursor mRNA into the third ventricle of male and female birds reduced resting time, spontaneous production of complex vocalizations, and stimulated brief agonistic vocalizations. GnIH RNAi further enhanced song production of short duration in male birds when they were challenged by playbacks of novel male songs. These behaviors resembled those of breeding birds during territorial defense. The overall results suggest that GnIH gene silencing induces arousal. In addition, the activities of male and female birds were negatively correlated with GnIH mRNA expression in the PVN. Density of GnIH neuronal fibers in the ventral tegmental area was decreased by GnIH RNAi treatment in female birds, and the number of GnRH neurons that received close appositions of GnIH neuronal fiber terminals was negatively correlated with the activity of male birds. In summary, GnIH may decrease arousal level resulting in the inhibition of specific motivated behavior, such as in reproductive contexts (Ubuka et al., 2012b).

As in birds, GnIH has been found in the brains of all mammalian species studied to date, including humans (Fukusumi et al., 2001; Ukena et al., 2002; Yoshida et al., 2003; Clarke et al., 2009; Ubuka et al., 2009a,b, 2012a; Kriegsfeld et al., 2010). As described previously, the cDNA for GnIH encodes two LPXRFamide peptides, RFRP-1 and RFRP-3, in mammals. Across mammals, administration of GnIH suppresses gonadotropin secretion (Kriegsfeld et al., 2006; Clarke et al., 2008; Kadokawa et al., 2009; Sari et al., 2009; Pineda et al., 2010a,b; Ubuka et al., 2012a). Most studies to date have applied RFRP-3 as the mammalian ortholog of avian GnIH (Clarke et al., 2008; Kadokawa et al., 2009; Pineda et al., 2010a). Our recent study further showed a strong suppressive effect of RFRP-1 on LH in Siberian hamsters (Ubuka et al., 2012a). Consistent with results for GnIH across mammalian species, ICV injections of the GnIH-R (GPR147) antagonist, RF9, result in rapid and sustained, dose-dependent increases in LH in male and female mice and rats (Pineda et al., 2010b) and the ewe (Caraty et al., 2012). Together, these findings suggest that RFRP-1 and/or -3 operate as GnIH in mammalian species. Whether RFRP-1 and -3 operate synergistically in mammals to inhibit HPG axis activity or whether each peptide dominates differentially across species remains an interesting avenue for further investigation.

In rodents, GnIH cell bodies are clustered in the dorsomedial nucleus of the hypothalamus (DMH) with extensive projections to hypothothamic and limbic structures (Johnson et al., 2007; Kriegsfeld et al., 2010; Ubuka et al., 2012a). In other species, such as sheep, GnIH neurons are located in both the PVN and more widely distributed throughout the mediobasal hypothalamus (Smith et al., 2008). With regard to reproduction, GnIH neurons have been shown to project to the external layer of the median eminence of Syrian hamsters, with the expression of GnIH-R (GPR147) observed in pituitary in the same investigation (Gibson et al., 2008). These findings suggested that, as in birds, GnIH may act at the pituitary level to suppress gonadotropin secretion (Figure 1). In sheep, GnIH administration down-regulates LHβ- and FSHβ-subunit expression (Sari et al., 2009) and results in marked reductions in LH pulse amplitude without affecting pulse frequency (Clarke et al., 2008). In cows, repeated IV administration of GnIH leads to reduced LH pulse frequency (Kadokawa et al., 2009), indicating the possibility of different modes of action across species. Taken together, these findings provide convincing evidence that the function of GnIH is conserved in mammals as well as in birds.

Smith et al. (2012) measured GnIH-3 (RFRP-3) concentrations in hypophyseal portal blood in ewes during the non-breeding (anestrous) season and during the luteal and follicular phases of the estrous cycle in the breeding season. Pulsatile GnIH-3 secretion was observed in the portal blood of all animals. Mean GnIH-3 pulse amplitude and pulse frequency were higher during the non-breeding season. GnIH-3 was virtually undetectable in peripheral blood plasma. To determine the role of secreted GnIH-3, they further examined its effects on GnRH-stimulated LH secretion in hypothalamo-pituitary-disconnected ewes, and found a significant reduction in the LH response to GnRH. GPR147 was expressed in fractions enriched for gonadotropes somatotropes, and lactotropes (Smith et al., 2012). IV administration of RF9 to anestrous acyclic ewes induced a sustained increase in LH plasma concentrations, and the increase in LH plasma concentrations induced by RF9 was blocked by previous administration of GnRH antagonist, Teverelix (Caraty et al., 2012). These data clearly show that GnIH-3 is secreted into portal blood to act on pituitary gonadotropes, reducing the action of GnRH in sheep.

Some disparity across studies and species has drawn into question the generality of the actions of GnIH directly on the pituitary. In one study in rats, for example, GnIH fibers were not detected in the external layer of the median eminence (Rizwan et al., 2009). In the same study, peripheral injections of the retrograde tracer, Fluorogold, did not label GnIH cell bodies whereas a majority of cells bodies were labeled following central injections. Finally, in this study, IV injections of GnIH did not affect basal LH concentrations, but GnRH-induced LH secretion was modestly suppressed. This latter finding is consistent with work by another group indicating that GnIH had minimal impact on LH secretion in cultured rat pituitary, but significantly suppressed GnRH-induced LH secretion *in vitro* (Murakami et al., 2008). Curiously, in this study, IV injections of GnIH led to gross reductions in LH at 120 min post injection whereas central injections did not impact LH concentrations. In another study of rats, intraperitoneal (IP) injections of the GnIH-R (GRP147) antagonist, RF9, led to modest increases in LH *in vivo*, whereas application of RF9 to pituitary cultures was without effect (Pineda et al., 2010b). In contrast, central injections of this receptor antagonist markedly stimulated LH release. Another study using ovariectomized female rats treated with a low dose of estrogen, found no effect of central GnIH injections on LH (Anderson et al., 2009). Together, these findings make it difficult to reconcile the specific impact of GnIH on the pituitary, at least in rats. The disparity across studies likely results from differences in the time course of sample collection, antibody, peptide, and tissue preparation, and other differences in experimental procedures and the reproductive state of the animals investigated.

GnIH-R (GPR147) couples to Gαi to inhibit AC (Hinuma et al., 2000). A recent study of ours using immortalized mouse gonadotrope cell line (LβT2 cells) has demonstrated that the inhibitory action of mouse GnIHs (RFRPs) on gonadotropin gene expression is mediated by an inhibition of AC/cAMP/cAMP-dependent protein kinase (PKA)-dependent extracellular signal-regulated kinase (ERK) pathway (Son et al., 2012). In the sheep, GnIH can inhibit both GnRH-induced intracellular Ca^2+^ increase and ERK phosphorylation, impacting GnRH-induced gonadotropin release and synthesis (Sari et al., 2009).

As in birds, GnIH neurons project monosynaptically to GnRH neurons (Johnson et al., 2007; Smith et al., 2008; Kriegsfeld et al., 2010) and GnRH neurons express the GnIH-R, GPR147 in mammals (Rizwan et al., 2012; Ubuka et al., 2012a). GPR74, which GnIH binds with lower affinity (Bonini et al., 2000), is not expressed on GnRH neurons (Rizwan et al., 2012). Direct application of GnIH inhibits neuronal firing in GnRH cells and this inhibitory action of GnIH persists when amino acid transmission is blocked, providing strong evidence for a direct inhibitory role of GnIH on the GnRH neuronal system (Ducret et al., 2009; Wu et al., 2009). Similarly, ICV administration of GnIH decreases the activational state of GnRH neurons (Anderson et al., 2009). Together, these findings, combined with those indicating suppressive action of central GnIH administration or stimulatory action of central RF9 administration on LH concentrations (Kriegsfeld et al., 2006; Anderson et al., 2009; Rizwan et al., 2009; Pineda et al., 2010a,b), point to a potent role for GnIH in GnRH inhibition (Figure 1).

In addition, vertebrate gonads are the sites of synthesis and action of GnIH. The gonadal GnIH system was first discovered in European starlings and Japanese quail (Bentley et al., 2008). GnIH-ir was observed in the interstitial and germ cells in the quail testis (Bentley et al., 2008). GnIH-ir was also observed in the columnar epithelial cells in the quail epididymis (Bentley et al., 2008). GnIH-R mRNA was localized by *in situ* hybridization in the germ cells and interstitial cells in the testis and columnar epithelial cells in the epididymis in quail (Bentley et al., 2008). *In situ* hybridization of GnIH in house sparrow testis produced a strong reaction product localized to interstitial cells, and GnIH-R to interstitial cells and secondary spermatocytes (McGuire and Bentley, 2010a). In female birds, GnIH-ir substance was observed in the thecal and granulose layers in starling ovary (Bentley et al., 2008). The presence of transcripts for GnIH and GnIH-R in the gonads and associated tissues, and the expression of GnIH and GnIH-R by specific cell types involved in steroid biosynthesis and gamete maturation indicates the likely importance of GnIH as a paracrine/autocrine regulator within reproductive tissues (for a review, see McGuire and Bentley, 2010a). In house sparrows, testosterone secretion from gonadotropin-stimulated cultured house sparrow testes was significantly decreased by the application of GnIH *in vitro* (McGuire and Bentley, 2010b). As is the case in birds, GnIH and its receptor are robustly expressed in Syrian hamster testis (Zhao et al., 2010), indicating that a role for GnIH in gonadal function may be common to all vertebrates. In hamsters, GnIH expression is confined to seminiferous tubules, with spermatocytes and spermatids expression the GnIH-R, GPR147, suggesting an important role in spermatogenesis. In macaque, GnIH is expressed in Leydig cells, Sertoli cells, spermatogonia, and spermatocytes (McGuire and Bentley, 2010b), suggesting roles in steroidogenesis, spermatogenesis, and sperm production. GnIH is also found in the granulosa cells of mouse ovarian follicles during proestrus and estrus and in the luteal cells during diestrus 1 and 2 (Singh et al., 2011a), and macaque granulosa cells and oocytes (McGuire and Bentley, 2010b). In mice, GnIH was closely associated with GnRH expression in mouse ovarian tissue (Singh et al., 2011b), suggesting that a coordinated balance between GnRH and GnIH activity plays an important role in follicular development and atresia throughout the ovulatory cycle. More recently, GnIH was shown to suppress gonadotropin-induced progesterone production in human granulosa cells (Oishi et al., 2012).

## Regulation of GnIH synthesis and release by photoperiods and melatonin in photoperiodic animals

Identification of the regulatory mechanisms governing GnIH synthesis and release is important in understanding the physiological role of the GnIH system. The mechanisms regulating GnIH synthesis have been investigated in birds. In general, the annual changes in pineal melatonin secretion drive the reproductive responses of photoperiodic mammals (Bronson, 1989). However, several studies suggested that melatonin is not responsible for changes in seasonal reproduction in birds (e.g., Wilson, 1991; Juss et al., 1993). Despite information to the contrary, there are data available on regulation of seasonal processes by melatonin, including but not limited to that of gonadal activity and gonadotropin secretion (Ohta et al., 1989; Bentley et al., 1999; Bentley and Ball, 2000; Guyomarc'h et al., 2001; Rozenboim et al., 2002). More recently there has been the suggestion that the avian hypothalamus can synthesize melatonin *de novo* (Kang et al., 2007). These researchers demonstrated the presence of tryptophan hydroxylase 1 and 5-HT N-acetyl-transferase, two key enzymes in melatonin biosynthesis, along with melatonin. This finding could explain the lack of effect of pinealectomy on the avian reproductive system; day length information could still be encoded by the hypothalamic melatonin system. Considering the inhibitory effects of GnIH on gonadotropin release and synthesis (Tsutsui et al., 2000; Ciccone et al., 2004; Osugi et al., 2004; Ubuka et al., 2006) and the inhibitory effects of short-day (SD) photoperiods, we manipulated melatonin levels in quail, a highly photoperiodic bird species, by removing sources of melatonin and investigating the action of melatonin on GnIH expression in the quail brain (Ubuka et al., 2005).

The pineal gland and eyes are the major sources of melatonin in quail (Underwood et al., 1984). Ubuka et al. (2005) found that pinealectomy combined with orbital enucleation (Px+Ex) decreased the expression of GnIH precursor mRNA and the mature GnIH peptide in the diencephalon. Melatonin administration to Px+Ex birds caused a dose-dependent increase in the expression of GnIH precursor mRNA and production of mature peptide (Ubuka et al., 2005). Furthermore, the expression of GnIH increased under SD (Ubuka et al., 2005), when the nocturnal duration of melatonin secretion increases (Cockrem and Follett, 1985). Mel_1c_, a melatonin receptor subtype, was expressed by GnIH-ir neurons in the PVN, thus indicating a direct action of melatonin on GnIH neurons (Ubuka et al., 2005). Finally, melatonin receptor autoradiography further revealed specific binding of melatonin in the PVN (Ubuka et al., 2005). Taken together, these data indicate that melatonin acts directly on GnIH neurons via its receptor to induce GnIH expression (Figure 1). This was the first demonstration in any vertebrate that melatonin can directly induce synthesis of a neuropeptide, and indicates that GnIH is capable of transducing photoperiodic information to the avian reproductive axis via changes in the melatonin signal (Figure 1).

Melatonin is likely a key factor controlling GnIH neural function. Therefore, we further investigated the role of melatonin in the regulation of GnIH release and the correlation of GnIH release with LH release in quail. Melatonin administration dose-dependently increased GnIH release from hypothalamic explants *in vitro* (Chowdhury et al., 2010). Furthermore, GnIH release was photoperiodically controlled in quail with diurnal changes negatively correlated with plasma LH concentrations (Chowdhury et al., 2010). GnIH release from hypothalamic explants in quail exposed to long-day (LD) photoperiods was greater in tissues collected during the dark period than during the light period (Chowdhury et al., 2010). Conversely, plasma LH concentrations decreased during the dark period. In contrast to LD, GnIH release from hypothalamic explants increased in quail under SD, when the duration of nocturnal secretion of melatonin increases (Chowdhury et al., 2010). These results indicate that melatonin plays a role in stimulating not only GnIH synthesis but also GnIH release, thus inhibiting plasma LH concentrations in quail (Figure 1).

Although it is well-established that GnRH-I controls reproductive function in birds (Sharp et al., 1990), no direct association between the gonadal regression observed during SD and a decrease in hypothalamic GnRH-I has been made in quail (Foster et al., 1988; Dawson et al., 2001). In Chowdhury et al. (2010), we are the first to show that the release of GnIH from hypothalamic explants in quail previously exposed to SD was increased, and this increase was associated with a decrease in plasma LH levels and gonadal regression. Because melatonin stimulates GnIH synthesis and release and GnIH inhibits gonadotropin synthesis and release in quail, it could be assumed that the gonadal regression occurring in birds exposed to SD is a direct consequence of the melatonin/GnIH system on circulating LH levels (Figure 1).

In addition, studies on other avian species with breeding strategies different from that of quail are required for us to determine whether or not the melatonin effects on GnIH we observe in quail are universal in photoperiodic avian species. Unlike quail, which terminate reproduction during decreasing day lengths, many passerine species terminate their reproductive activities while day lengths are still increasing, so in those species presumably the effects of melatonin on GnIH synthesis and release would also be decreasing. There is also some evidence in sparrow species that the GnIH system is active during the breeding season (LD, short duration of melatonin secretion) and is responsible for the temporary cessation of reproductive activities in response to environmental stimuli such as stress (Bentley et al., 2003; Calisi et al., 2011). How the regulation of GnIH by melatonin fits into the seasonal reproductive profile of these species has not yet been determined.

In mammals, GnIH also appears to play an important role in monitoring internal and external status and integrating this information to control reproductive functioning precisely and maximize reproductive success. In hamsters, reproductive status is primarily driven by day lengths (photoperiods). If GnIH is involved in the control of reproductive functions in hamsters, the expressions of GnIH precursor mRNA and GnIH peptides should be regulated by photoperiod and melatonin. We found that expression of GnIH precursor mRNA, GnIH-immunoreactivity in GnIH-ir perikarya and GnIH-ir fiber density decreased under SD photoperiod compared to LD in Siberian hamsters, a highly photoperiodic mammalian species (Ubuka et al., 2012a). This inhibitory effect of SD was not seen if the hamsters were pinealectomized, and melatonin administration inhibited the expressions of GnIH precursor mRNA, GnIH-immunoreactivity in GnIH-ir perikarya and GnIH-ir fiber density. It was further shown that the percentage of GnRH-ir neurons receiving GnIH-ir fiber terminals decreased in SD photoperiod. These results are in line with the results in Syrian hamsters (Revel et al., 2008; Mason et al., 2010) and sheep (Dardente et al., 2008). These findings are consistent with the possibility that the activity of GnIH neurons decreases in SD by the inhibitory action of pineal melatonin in hamsters. The functional significance of this counterintuitive relationship between photoperiod and GnIH is not straightforward and is discussed further below. On the other hand, it was shown that melatonin directly induces GnIH mRNA expression in the rat GnIH cell line (rHypoE-7) (Gingerich et al., 2009). These results demonstrate that the expression of GnIH is photoperiodically modulated via a melatonin-dependent process in mammals, like in birds, although how it is related to seasonal reproduction is not yet clear (Figure 1).

There are different actions of melatonin on GnIH expression across species (Figure 1). Melatonin increases the expression of GnIH in quail, a photoperiodic bird, (Ubuka et al., 2005; Chowdhury et al., 2010). A similar, but opposite, action of melatonin on the inhibition of the expression of GnIH was shown in photoperiodic mammals, such as Syrian and Siberian hamsters (Revel et al., 2008; Mason et al., 2010; Ubuka et al., 2012a) and sheep (Dardente et al., 2008). Quail is a LD breeder, which activates its reproductive activity in LD and suppresses its reproductive activity in SD. It is understandable that the expression of GnIH is stimulated by melatonin, a nocturnal hormone, and SD when the duration of melatonin secretion is long. Accordingly, it was hypothesized that the increase of GnIH expression may inhibit reproductive activity in SD in quail (Ubuka et al., 2005). The opposite but similar thoughts can be applied to sheep. Sheep is a SD breeder that activates its reproductive activity in SD and suppresses its reproductive activity in LD. It is also understandable that the expression of GnIH is inhibited by melatonin, a nocturnal hormone, and SD when the duration of melatonin secretion is long. In contrast, the expression of GnIH increases in LD in sheep. Accordingly, it was hypothesized that the increase of GnIH expression may inhibit reproductive activity in LD in sheep (Dardente et al., 2008). On the other hand, it was difficult to understand the inhibitory effect of melatonin or SD on GnIH expression in hamsters, because hamsters are LD breeders (Revel et al., 2008; Mason et al., 2010). Some clarity was garnered from a recent report showing that GnIH may have a stimulatory effect on gonadotropin secretion in SD in Siberian hamsters (Ubuka et al., 2012a). Long duration of melatonin secretion in SD may need to decrease GnIH expression to inhibit reproductive activities of Siberian hamsters in SD. Recently, Ancel et al. (2012) reported stimulatory effects of RFRP-3 on the gonadotrophic axis in the male Syrian hamster (Ancel et al., 2012), underscoring the importance of considering sex and photoperiodic conditions when evaluating the role of GnIH. Further studies are required to understand the role of melatonin controlling GnIH expression in photoperiodic animals (see the section of Neuroendocrine Integration of Environmental and Internal Signals).

Several lines of evidence indicate that kisspeptin and GnIH might work in concert to guide seasonal changes in reproduction across species (reviewed in Simonneaux et al., 2012). In Syrian hamsters, for example, Kiss1 mRNA levels are lower in the Arc of SD hamsters and melatonin appeared to be necessary for the seasonal change; pineal-gland ablation prevents the SD-induced down-regulation of Kiss1 expression (Revel et al., 2006). In Syrian hamsters, short day lengths lead to decreased kisspeptin-ir in the anteroventral periventricular nucleus (AVPV) and increased expression in the Arc (Greives et al., 2007). In Siberian hamsters, chronic administration of kisspeptin restored testicular activity of SD hamsters despite persisting photoinhibitory conditions. The authors propose that photoperiod, via melatonin, modulates Kiss1 signaling to drive the reproductive axis (Revel et al., 2006). In Syrian hamsters, chronic administration of kisspeptin does not restore reproductive competence to SD animals, likely due to downregulation of kisspeptin receptor (Greives et al., 2008). Data from these studies suggest that GnIH and kisspeptin may both be direct or indirect targets of melatonin, decoding photoperiod, and appropriately signaling the reproductive axis based on time of year.

## Effect of stress on GnIH synthesis

Calisi et al. (2008) hypothesized that suppressive effects of stress upon reproduction are mediated by the hypothalamic GnIH system in birds. The effects of capture-handling stress on the numbers of GnIH neurons in the hypothalamus of adult male and female house sparrows were tested. There were more GnIH-positive cells in birds in fall vs. those sampled in the spring, and a significant increase in GnIH positive cells was seen in stressed birds in spring. These data imply an influence of stress upon the GnIH system. It is likely that this effect of immobilization stress is mediated directly via glucocorticoid action on GnIH neurons via glucocorticoid receptor (GR).

Kirby et al. (2009) also investigated how acute immobilization stress alters GnIH mRNA and protein levels in adult male rats. GnIH mRNA and protein levels increased immediately following stress but returned to a lower level 24 h after stress. Thus, GR expression by GnIH neurons is evident in birds and mammals, and is likely to be an evolutionarily conserved phenomenon. GnIH may therefore be a mediator of stress-induced reproductive disruption in birds and mammals (Figure 1). The role of the GnIH system in suppressing reproduction during times of stress may not be common across species; stress does not affect GnIH peptide or mRNA expression in sheep (Papargiris et al., 2011).

## Neuroendocrine integration of environmental and internal signals

In birds, termination of breeding is coincident with long-duration melatonin secretion in the fall. Although the termination of breeding results from refractoriness to melatonin stimulation, given the actions on melatonin on GnIH (Ubuka et al., 2005; Chowdhury et al., 2010), it is likely that this neuropeptide is involved in avian reproductive termination in response to the nocturnal melatonin signal. More recently, melatonin implants have been shown to delay the onset of clutch initiation in female great tits but without affecting clutch size, body mass, or timing of onset of activity (Greives et al., 2012). In other species, GnIH and GnRH expression are positively correlated (Bentley et al., 2003; Calisi and Bentley, 2009). Such a relationship may appear counter-intuitive: why should an inhibitory hormone increase during the breeding season (Dawson et al., 2001)? We hypothesize that in some species, GnIH acts to induce a temporary pause in reproductive effort without full HPG axis suppression and regression, which would cause the individual to miss the opportunity to breed in that year. Such a missed opportunity in a single year could markedly reduce reproductive success in short-lived songbirds. In their natural environments, this temporary cessation of reproduction occurs in response to unpredictable environmental cues, such as stress, or mate/nest availability. Some support for this possibility comes from an experiment on European starlings in which nesting opportunities were limited (Calisi et al., 2011). Under these conditions, birds that outcompeted others for nest boxes (“winners”) had significantly fewer numbers of GnIH-producing cells than those without nest boxes (“losers”) at the beginning of the breeding season (Calisi et al., 2011). This relationship changed with breeding stage; once “winners” had begun to incubate eggs, their GnIH content increased significantly above that of “losers.” Thus, whereas birds appeared reproductively capable across treatments, these findings indicate that hypothalamic GnIH may serve as a short-term modulator of reproductive behaviors in response to social environment. Consequently, hypothalamic GnIH may serve as a regulator of reproductive behaviors in response to social environment (Calisi et al., 2011). How this pattern of GnIH expression during LD in a naturally-housed songbird relates to the increased expression and release of GnIH by SD-induced melatonin action in laboratory quail (Ubuka et al., 2005; Chowdhury et al., 2010) remains to be seen. Studies on wild quail might provide some valuable answers to this question (see Calisi and Bentley, 2009).

As in birds, mammals in their natural environments are faced with the challenge of maximizing reproductive success while considering obstacles to survival such as reduced food availability and inclement weather (Bronson, 1989). As a result, animals must have a mechanism for transducing environmental conditions into neural signals to guide sexual motivation and reproductive axis activity. The GnIH system is well-situated to act as an intermediary between the environment and the reproductive axis. For example, GnIH neurons project to neuropeptide-Y, pro-opiomelanocortin, orexin and melanin concentrating cells in the ovine brain (Clarke et al., 2009; Qi et al., 2009), providing a neural circuit capable of driving motivation to feed over sex during unfavorable energetic conditions. Consistent with this notion, administration of GnIH increases feeding across mammalian species (Johnson et al., 2007). Likewise, in Syrian hamsters, NPY cells project to GnIH cells in the DMH, providing a neural pathway for interpretation of energetic status and initiation of GnIH-induced inhibition of reproductive function during times of reduced food availability (Klingerman et al., 2011). In agreement with this possibility, GnIH neuronal activity is increased following food deprivation in this species (Klingerman et al., 2011).

## Conclusion

The discovery of GnIH has fundamentally changed our understanding of hypothalamic control of reproduction. Recent studies on photoperiodic birds and mammals have demonstrated that GnIH expression is photoperiodically modulated via a melatonin-dependent process. Melatonin, a nocturnal hormone, appears to act directly on GnIH neurons through its receptor to induce expression and release of GnIH in quail and rats. A similar, but opposite, action of melatonin on the inhibition of expression of mammalian GnIH is found in hamsters and sheep. The variety of melatonin actions and modes of regulation of GnIH raise interesting questions as to the evolution of GnIH and its adaptive significance. Recent studies have further demonstrated that stress induces GnIH expressions in birds and mammals. GnIH may therefore be a mediator of stress-induced reproductive disruption. Thus, the neuroendocrine integration of environmental signals, such as photoperiods and stress, and internal signals, such as GnIH, melatonin, and glucocorticoids, is important for the control of avian and mammalian reproduction.

### Conflict of interest statement

The authors declare that the research was conducted in the absence of any commercial or financial relationships that could be construed as a potential conflict of interest.
